# Supplementary material for: A faster and less costly alternative for RNA extraction of SARS-CoV-2 using proteinase k treatment followed by thermal shock
Source: PLoS One. 2021 Mar 24;16(3):e0248885. doi: 10.1371/journal.pone.0248885 (PMC7990203; doi:10.1371/journal.pone.0248885)
Supplement: S1 Appendix — (DOCX) [file pone.0248885.s001.docx]

**S1 Appendix: Real time RT-PCR multiplex for SARS-CoV-2 using GAPDH as internal control**

1. **Primers, probes, and targets for amplification**

| Target | Primers (F or R)  Probes (P or P2) | Sequence 5’ → 3’ | Reference |
| --- | --- | --- | --- |
| *RdRp*  *(SARS-CoV-2)* | *RdRp_SARSr-F* | GTGARATGGTCATGTGTGGCGG | Corman et al., 2020 |
|  | *RdRp_SARSr-P2* | FAM-CAGGTGGAACCTCATCAGGAGATGC-BBQ |  |
|  | *RdRp_SARSr-R* | CARATGTTAAASACACTATTAGCATA |  |
| *GAPDH*  *(Human)* | *GAPDH-F* | GTGAAGGTCGGAGTCAACGG | Thongpan et al., 2019 |
|  | *GAPDH-P* | ROX-CGCCTGGTCAACAGGGTCGC-BBQ |  |
|  | *GAPDH-R* | TCAATGAAGGGGTCATTGATG |  |

1. **Reactions’ conditions**

| Reagents | Concentration | Volume (µL) |
| --- | --- | --- |
| CAPITALTM 1-step qRT-PCR Probe Mix* | 4x | 5.0 |
| Water* | --- | 5.6 |
| RdRp_SARSr-F | 10.00µM | 0.8 |
| RdRp_SARSr-P2 | 10.00 µM | 0.4 |
| RdRp_SARSr-R | 10.00 µM | 0.8 |
| GAPDH-F | 2.50 µM | 0.5 |
| GAPDH-P | 1.25µM | 0.4 |
| GAPDH-R | 2.50 µM | 0.5 |
| RNAse inhibitor | 1x | 1.0 |
| RNA | --- | 5.0 |
| Final Volume | --- | 20.0 |

*These values (concentration and volume) can change according to enzyme that to be used in the reactions, which must follow the manufacturer's guidelines.

1. **Enzyme and thermo-cycling conditions**

| **Step** | **Enzyme** | **Temperature** | **Time** | **Number of cycles** |
| --- | --- | --- | --- | --- |
| **Reverse transcription** | Biotechrabbit | 50°C | 10 minutes | 1 |
|  | Invitrogen | 50°C | 30 minutes | 1 |
|  | Qiagen | 50°C | 15 minutes | 1 |
| **Denaturation** | Biotechrabbit | 95 °C | 3 minutes | 1 |
|  | Invitrogen | 95 °C | 2 minutes | 1 |
|  | Qiagen | 95 °C | 5 minutes | 1 |
| **Amplification** | Biotechrabbit | 95 °C | 10 seconds | 45 |
|  |  | 58 °C | 30 seconds* |  |
|  |  | 40 °C | 30 seconds |  |
|  | Invitrogen | 95 °C | 15 seconds | 45 |
|  |  | 58 °C | 30 seconds |  |
|  |  | 40 °C | 30 seconds |  |
|  | Qiagen | 95 °C | 15 seconds | 45 |
|  |  | 58 °C | 30 seconds |  |
|  |  | 72 °C | 15 seconds |  |

1. **Analysis and interpretation of results**

The curves were analyzed considering the *RdRp* gene channel (Green, 470 nm to excitation, 510 nm to detection, for being the probe marked with FAM fluorescent) and the *GAPDH* gene channel (Orange, 585 nm to excitation, 610 nm to detection for being the probe marked with ROX fluorescent). The cycle threshold (Ct) values ≤ 37 were reported as positive samples for the SARS-CoV-2 RdRp gene and those with Ct >37 for the same gene and an internal control Ct ≤ 40. Samples with internal control Ct > 40 were no considered (degraded sample).

1. **References**
2. Corman VM, Landt O, Kaiser M, Molenkamp R, Meijer A, Chu DK, Bleicker T, Brünink S, Schneider J, Schmidt ML, Mulders DG, Haagmans BL, van der Veer B, van den Brink S, Wijsman L, Goderski G, Romette JL, Ellis J, Zambon M, Peiris M, Goossens H, Reusken C, Koopmans MP, Drosten C. Detection of 2019 novel coronavirus (2019-nCoV) by real-time RT-PCR. Euro Surveill. 2020 Jan;25(3):2000045. doi: 10.2807/1560-7917.ES.2020.25.3.2000045. Erratum in: Euro Surveill. 2020 Apr;25(14): Erratum in: Euro Surveill. 2020 Jul;25(30): PMID: 31992387; PMCID: PMC6988269.
3. Thongpan I, Suntronwong N, Vichaiwattana P, Wanlapakorn N, Vongpunsawad S, Poovorawan Y. Respiratory syncytial virus, human metapneumovirus, and influenza virus infection in Bangkok, 2016-2017. PeerJ. 2019 Apr 11;7:e6748. doi: 10.7717/peerj.6748. PMID: 30997293; PMCID: PMC6462397.
