# Supplementary material for: A faster and less costly alternative for RNA extraction of SARS-CoV-2 using proteinase k treatment followed by thermal shock
Source: PLoS One. 2021 Mar 24;16(3):e0248885. doi: 10.1371/journal.pone.0248885 (PMC7990203; doi:10.1371/journal.pone.0248885)
Supplement: S1 File — (DOCX) [file pone.0248885.s002.docx]

**EXTRACCIÓN DE ARN POR SHOCK TÉRMICO PARA LA**

**RT- PCR MULTIPLEX EN TIEMPO REAL PARA LA DETECCIÓN DEL VIRUS SARS-CoV-2**

**MET-CNSP-196**

| **Elaborado por:** | CNSP Blgo. Adolfo Ismael Marcelo Ñique  CNSP Blga. Johanna Nery Balbuena Torres |
| --- | --- |
| **Revisado por:** | CNSP Blga. Nancy Rojas Serrano (Revisión técnica).  CNSP Blga. Gloria Arotinco Garayar (Revisión formal). |
| **Aprobado por:** | CNSP Méd. Lely del Rosario Solari Zerpa |
| RD N° -2020-DG-CNSP/INS Fecha: / / 2020 | |

**ÍNDICE**

**Pág**

1. ÁMBITO DE APLICACIÓN 3

2. REFERENCIAS 3

3. DEFINICIONES OPERATIVAS 3

4. FUNDAMENTO DEL MÉTODO 4

5. DESARROLLO DEL MÉTODO DE ENSAYO 4

6. CÁLCULOS 9

7. INFORME DE RESULTADOS 10

8. INTERPRETACIÓN DE RESULTADOS 10

9. FORMULARIOS 10

11. CONTROL DE CAMBIOS 10

12. ANEXOS 10

1. **ÁMBITO DE APLICACIÓN**

El método de ensayo se ejecuta en el Laboratorio Referencia Nacional de Virus Respiratorios del Centro Nacional de Salud Pública del INS, donde es aplicado a muestras humanas respiratorias de hisopado nasal y faríngeo, para la detección *in vitro* del genoma del virus SARS-CoV-2, mediante shock térmico.

1. **REFERENCIAS**

2.1. [Corman Victor M](https://www.eurosurveillance.org/search?value1=Victor+M+Corman&option1=author&noRedirect=true), [Landt Olfert](https://www.eurosurveillance.org/search?value1=Olfert+Landt&option1=author&noRedirect=true), [Kaiser Marco](https://www.eurosurveillance.org/search?value1=Marco+Kaiser&option1=author&noRedirect=true), [Molenkamp Richard](https://www.eurosurveillance.org/search?value1=Richard+Molenkamp&option1=author&noRedirect=true), [Meijer Adam](https://www.eurosurveillance.org/search?value1=Adam+Meijer&option1=author&noRedirect=true), [Chu Daniel KW](https://www.eurosurveillance.org/search?value1=Daniel+KW+Chu&option1=author&noRedirect=true), [Bleicker Tobias](https://www.eurosurveillance.org/search?value1=Tobias+Bleicker&option1=author&noRedirect=true), [Brünink Sebastian](https://www.eurosurveillance.org/search?value1=Sebastian+Br%C3%BCnink&option1=author&noRedirect=true), [Schneider Julia](https://www.eurosurveillance.org/search?value1=Julia+Schneider&option1=author&noRedirect=true), [Schmidt Marie Luisa](https://www.eurosurveillance.org/search?value1=Marie+Luisa+Schmidt&option1=author&noRedirect=true), [Mulders Daphne GJC](https://www.eurosurveillance.org/search?value1=Daphne+GJC+Mulders&option1=author&noRedirect=true), [Haagmans Bart L](https://www.eurosurveillance.org/search?value1=Bart+L+Haagmans&option1=author&noRedirect=true), [van der Veer Bas](https://www.eurosurveillance.org/search?value1=Bas+van+der+Veer&option1=author&noRedirect=true), [van den Brink Sharon](https://www.eurosurveillance.org/search?value1=Sharon+van+den+Brink&option1=author&noRedirect=true), [Wijsman Lisa](https://www.eurosurveillance.org/search?value1=Lisa+Wijsman&option1=author&noRedirect=true), [Goderski Gabriel](https://www.eurosurveillance.org/search?value1=Gabriel+Goderski&option1=author&noRedirect=true), [Romette Jean-Louis](https://www.eurosurveillance.org/search?value1=Jean-Louis+Romette&option1=author&noRedirect=true), [Ellis Joanna](https://www.eurosurveillance.org/search?value1=Joanna+Ellis&option1=author&noRedirect=true), [Zambon Maria](https://www.eurosurveillance.org/search?value1=Maria+Zambon&option1=author&noRedirect=true), [Peiris Malik](https://www.eurosurveillance.org/search?value1=Malik+Peiris&option1=author&noRedirect=true), [Goossens Herman](https://www.eurosurveillance.org/search?value1=Herman+Goossens&option1=author&noRedirect=true), [Reusken Chantal](https://www.eurosurveillance.org/search?value1=Chantal+Reusken&option1=author&noRedirect=true), [Koopmans Marion PG](https://www.eurosurveillance.org/search?value1=Marion+PG+Koopmans&option1=author&noRedirect=true), [Drosten Christian](https://www.eurosurveillance.org/search?value1=Christian+Drosten&option1=author&noRedirect=true). Detection of 2019 novel coronavirus (2019-nCoV) by real-time RT-PCR. [Euro Surveill.](https://www.eurosurveillance.org/content/ecdc) 2020;25(3):pii=2000045.

2.2 EXT-CNSP-007: BD Universal Viral transport.

2.3 EXT-CNSP-673: Copan universal Transport medium system (UTM-RT).

2.4 EXT-CNSP-766: Capital ^TM^ qRT-PCR Probe Mix, 4X.

2.5 EXT-CNSP-862: QIAamp Viral RNA Mini Handbook for Purification of viral RNA from plasma, serum, cell-free body fluids and culture supernatants March 2018.

2.6 EXT-CNSP-863: REMEL Micro Test TM M4RT Medio multimicrobiano.

2.7 EXT-CNSP-953: Viral Nucleic Acid Extraction Kit II, Geneaid.

2.8 EXT-CNSP-955: Rotor Gene Multiplex RT-PCR Kits (400), Qiagen.

2.9 EXT-CNSP-959: Proteinasa K.

2.10 EXT-CNSP-960: Natacha Merindola, Geneviève Pépinc, Caroline Marchand, Marylène Rheaultb, Christine Peterson, André Poirierd, , Claudia Houled, Hugo Germaina, Alexis Danylod. SARS-CoV-2 detection by direct rRT-PCR without RNA extraction.

# **DEFINICIONES OPERATIVAS**

Carrier: ARN de pequeño tamaño que es componente de algunos kits de purificación de ARN, el cual permiten mejorar la eficiencia de recuperación del ARN de la muestra.

Control Interno: amplificación de ARN humano producido por un gen que se exprese de manera constante. Por ejemplo, RNAsaP humana.o GAPDH.

Control Positivo: ARN positivo de referencia o preparados en el laboratorio.

Control Negativo: ARN a partir de hisopados nasal y faríngeos de personas clínicamente sanas

Cycle Threshold o Ciclo Umbral: Es el número de ciclos necesarios para que se produzca un aumento de fluorescencia significativo con respecto a la señal de base, y es inversamente proporcional a la cantidad inicial de moléculas molde. Se calcula en escala logarítmica.

Línea de base o baseline: Ciclos iniciales del PCR, donde no hay cambios significativos en la señal de fluorescencia. Determina la fluorescencia basal.

NTC: siglas de “No Template Control”, tubo de reacción utilizado como control que contiene la mezcla de reacción completa excepto de ácidos nucleicos (ARN o ADN) o molde para la amplificación.

Primer o cebador: Es una secuencia corta de ácido nucleico que contiene un grupo 3' hidroxilo libre que forma pares de bases con una hebra molde complementaria y actúa como punto de inicio para la adición de nucleótidos con el fin de copiar la hebra molde. Se necesitan dos primer para la PCR.

PCR en tiempo real: Es una variación de la técnica PCR estándar o convencional para la cuantificación de DNA/RNA en una muestra. Que Consta de 3 fases: Desnaturalización, hibridación y elongación. La detección se realiza en el termociclador mediante la emisión de fluorescencia midiendo la tasa de generación de productos.

RT-PCR: Es la PCR de transcriptasa reversa, en el cual existe el paso de retro transcripción de ARN a ADN complementario.

SIGLAS

CT : Cycle Threshold o Ciclo Umbral.

EPP : Equipo de Protección Personal.

RT-PCR en tiempo real: transcripción reversa acoplada PCR por tiempo real.

# **FUNDAMENTO DEL MÉTODO**

La transcripción reversa acoplada a la reacción en cadena de la polimerasa en tiempo real (RT-PCR), permite el uso de ARN como molde, la detección y amplificación del ARN de coronavirus SARS-COV2.

El ARN se transcribe de forma inversa a ADN complementario (cADN) utilizando la enzima transcriptasa inversa, luego este cADN es amplificado por la enzima ADN polimerasa termoestable durante los ciclos de amplificación, esta amplificación se detecta inmediatamente mediante la cinética de acumulación de amplicones a cada ciclo la cual se evidencia por la fluorescencia emitida en el tubo de reacción por una molécula reportera.

# **DESARROLLO DEL MÉTODO DE ENSAYO**

## **Aspecto de bioseguridad**

La prueba de RT-PCR en tiempo real se realiza en el laboratorio de virus respiratorio en ambientes de Nivel de Bioseguridad II, según lo detallado en el **ITT-CNSP-455: Instrucciones de bioseguridad para el laboratorio de microbiología y biomedicina del centro nacional de salud pública.**

Para el desarrollo de esta técnica se deben de tomar en cuenta las siguientes consideraciones:

- Mantener áreas separadas: lugar de extracción de ARN (LVR-NBS-II) el lugar de preparación de los reactivos de la mezcla de reacción para el PCR (Área Limpia- LBBM) y el lugar donde se adicionará el ARN extraído (LVR-NBS-II).

## **Tipos de muestras**

### Muestras primarias

#### Hisopado nasal y faríngeo.

#### Considerar otros aspectos de acuerdo al ITT-CNSP-385: Toma, manejo y envió de muestras para el diagnóstico de virus respiratorios.

### Material de referencia

**Controles Positivos:**

**Controles (OPS***)*: Controles positivos de coronavirus SARS-CoV-2

- 1. **Equipo e insumos requeridos (trazabilidad metrológica)**

### Equipos / instrumentos

- Cabina de bioseguridad clase IIA.
- Cabina de flujo laminar para PCR.
- Termociclador para PCR en tiempo real (RotorGene Q®, CFX96 Biorad® y Line Gene BIOER 9660®)
- Computadora acoplada al termociclador de PCR en tiempo real.
- Termociclador convencional
- Equipo automatizado para extracción de RNA.
- Microcentrífuga no refrigerada.
- Refrigeradora 2 a 8 ^o^C.
- Congelador ^–^ 20 ^o^C.
- Congelador ^–^ 80 ^o^C.
- Vortex.
- Micropipeta de 0.5 – 10 µL.
- Micropipeta de 20 - 200 µL.
- Micropipeta de 100 -1000 µL.
- Micropipeta de 2 – 20 µL
- Micropipeta multicanal de 0.5 – 10 µL.

### Insumos

#### Materiales

- Puntas con filtro estériles de 0.5 a 10 µL.
- Puntas con filtro estéril de 20 - 200 µL.
- Puntas con filtro estéril de 2-20 µL.
- Puntas con filtro estéril de 1000 µL.
- Gradilla para tubos de 5 mL
- Gradilla para tubos de 1.5 mL.
- Microplacas de 96 pocillos.
- Tubos para centrifuga de 1.5 mL libres de ARNasas y ADNasas.
- Tubos de 2 mL para viroteca
- Placa para tubos de PCR 0.2 mL refrigeradas.
- Cajas Criogénicas.
- Parafilm.
- Tubos de polipropileno para PCR en tiempo real (0.2 mL ).
- Placa de ópticamente transparente para PCR en tiempo real (0.2 mL ).
- film adhesivo para placas de PCR

#### Reactivos

- Kit Enzima transcriptasa reversa/ADN polimerasa para RT-PCR en tiempo real.
- Sondas y Primers.
- Agua destilada ultra pura de grado molecular (libre de RNAsa y DNasa).
- Etanol grado biología molecular
- Proteinasa K

#### Área de trabajo

- Área de Virus respiratorio (Nivel de Bioseguridad II).
- Área limpia de biología molecular (Nivel de Bioseguridad II).
  1. **Especificaciones técnicas:** Las especificaciones técnicas de la prueba.
     1. Criterios de la prueba:

Negativo: Valor Ct > 37.

Positivo: Valor Ct ≤ 37.

## **Interferencias y reacciones cruzadas**

### Muestras que no se encuentran en cadena de frio.

- Muestras contaminadas.
- Muestras con hisopos de madera o alginato.
- Condiciones Ambientales (amplicones, polvo).

## **Fuentes potenciales de variabilidad**

### Almacenamiento inadecuado de reactivos.

### Desperfecto en el funcionamiento de los equipos: termociclador, extractor de ácidos nucleicos.

### Inadecuada preparación de reactivos: buffer de lisis, controles positivos, control negativo, control humano y mezcla de rRT-PCR.

## **Condiciones previas**

### Preparación de reactivos y materiales

1. Realizar la limpieza de superficies de trabajo, micropipetas y termocicladores con productos como: alcohol 70% o solución comercial que elimine contaminantes, como ácido nucleico para minimizar el riesgo de contaminación.
2. **La cabina de bioseguridad**, antes de iniciar la extracción de ARN debe ser limpiada con solución de descontaminación comercial o alcohol del 70% y colocar los materiales mínimos necesarios y pipetas a ser utilizadas e irradiar con luz ultravioleta durante 15 minutos.
3. **La cabina de PCR del área limpia,** antes de iniciar la preparación de la mezcla de reacción se debe limpiar con solución de descontaminación comercial o alcohol del 70% e irradiar con luz ultravioleta durante 15 minutos. con la finalidad de elimine contaminantes.
4. **La cabina para cargar el ARN,** debe ser limpiada con solución de descontaminación comercial o alcohol del 70% y colocar las puntas para micropipetas, las micropipetas a ser utilizadas e irradiar con luz ultravioleta durante 15 minutos.

**Nota. -** Esto se realizará cada vez que se procese las muestras.

1. El termociclador será limpiado después de su uso con solución de descontaminación (alcohol a 70% o alguna solución comercial que elimine contaminantes).
2. Los reactivos deben ser previamente descongelados y conservados en cadena de frio.
3. En el caso de las enzimas mantener en congelación (-20°C).

### Otros

- Mantener temperatura ambiental del laboratorio entre 19 a 25 °C.
- Verificar el funcionamiento de los equipos.

## **Procedimiento**

Utilizar el **FOR-CNSP-104: Protocolo de trabajo de extracción de ADN/ARN de virus influenza y otros virus respiratorios** para registrar los códigos de las muestras, **FOR-CNSP-590: Registro de resultados para otros vírus respiratórios** y el **FOR-CNSP-095: Control de uso de equipos.**

- - 1. **Extracción manual de ARN viral utilizando Shock Térmico y Proteinasa K:**

El procedimiento de extracción se realiza en el ambiente de virus respiratorios Nivel de Bioseguridad II.

- Homogenizar la muestra con el agitador de tubos por 10 a 15 segundos.
- Alicuotar 100 µL de la muestra en placas de PCR o tubos de 0.2 mL.
- Agregar 3 µL de Proteinasa K en solución a cada muestra.
- Colocar el termociclador convencional, bajo las siguientes condiciones:

1 ciclo 56°C x 10 min

98°C x 05 min

4°C x 2 min

- Realizar una centrifugación breve para asegurarse de que el contenido este en el fondo de los pocillos.
- El proceso de RT-PCR tiempo real fue el mismo tanto para obtenido por el método convencional de extracción, como del extraído por Shock térmico/PK.
- El ARN extraído se puede almacenar en el refrigerador de 2 a 8 °C hasta continuar con la prueba molecular de PCR.
  - 1. **Preparación de la mezcla de reacción (MIX):**
       1. La preparación de la mezcla de reacción se realiza en el área limpia del laboratorio de Biotecnología y Biología Molecular (LBBM-NBS-II).
       2. Si es necesario reconstituir los primers y sondas liofilizadas (ver Anexo 01). Si los primers y sondas ya están preparados utilizar la alícuota correspondiente.
       3. Homogenizar los componentes y proceder a la preparación de la mezcla de reacción.
       4. Determinar el número de reacciones necesarias considerando el número de muestras, el control NTC (utilizar agua pura libre de nucleasas), control negativo (gen humano) y el control positivo (CP).
       5. Rotular un vial de 1.5 mL como mezcla de reacción de RdRP y otro con GAPDH.
       6. Distribuir los reactivos según el orden que se indica a continuación (de acuerdo al reactivo a usar):

1. **Preparación de la mezcla de reacción multiplex – protocolo Berlín - usando kit comercial de la marca QIAGEN**

Debido a la necesidad de evaluar otras marcas comerciales se estandarizo y evaluó la preparación de la mezcla de reacción de RT-PCR en tiempo real en formato multiplex utilizando el kit comercial Rotorgene multiplex RT-PCR de la marca QIAGEN.

- Preparar los tubos de reacción en el área limpia usando la siguiente formulación (considerar una cantidad de reacciones suficiente para incluir las muestras, los controles NTC, control negativo, control positivo y un tubo de exceso):

| **Reactivos** | **Volumen por Muestra (1X)** µL |
| --- | --- |
| Agua para PCR | 1.35 |
| PCR Mix Buffer (2X) | 10 |
| Primer forward gen RdRP, 10 µM | 0.8 |
| Primer reverse gen RdRP, 10 µM | 0.8 |
| Sonda gene RdRP, 10 µM | 0.4 |
| Primer forward GAPDH, 25 µM | 0.5 |
| Primer reverse GAPDH, 25 µM | 0.5 |
| Sonda GAPDH 12.5 µM | 0.4 |
| Mezcla de RT/Taq | 0.25 |
| **TOTAL DE MEZCLA DE REACCIÓN** | **15** |

- Repartir 15 µL de esta mezcla de reacción en tubos de PCR de 0.1 mL.
- En el área de procesamiento añadir 5 µL de ARN purificado.
- Correr las siguientes condiciones en el termociclador para RT-PCR en tiempo real.

| Transcripción reversa | 50°C X 15 min |
| --- | --- |
| Activación de la polimerasa | 95°C x 5 min |
| Amplificación  45 ciclos | 95°C x 15 seg |
|  | **58°C x 30 seg** |
|  | 72°C x 15 seg |

Adquirir en la etapa de hibridación en los canales Green y Orange a 58°C. Utilizar la opción de optimización de la fluorescencia durante el primer ciclo.

1. **Preparación de la mezcla de reacción multiplex – protocolo Berlín - usando kit comercial de la marca Biotechrabbit Capital ^TM^ qRT-PCR Probe Mix, 4X.**

| **Reactivos** | **Volumen por Muestra (1X)** µL |
| --- | --- |
| Agua para PCR | 5.6 |
| PCR Mix Buffer (2X) | 5.0 |
| Primer forward gen RdRP, 10 µM | 0.8 |
| Primer reverse gen RdRP, 10 µM | 0.8 |
| Sonda gene RdRP, 10 µM | 0.4 |
| Primer forward GAPDH, 25 µM | 0.5 |
| Primer reverse GAPDH, 25 µM | 0.5 |
| Sonda GAPDH 12.5 µM | 0.4 |
| Enzima Rtase con inhibator | 1 |
| **TOTAL DE MEZCLA DE REACCIÓN** | **15** |

- - - 1. Colocar los tubos de PCR 0.1 mL, en la placa de aluminio refrigerada y distribuir 15 μL del mix en los tubos.
      2. Agregar 5 μL agua de PCR en el primer tubo, tapar todos los tubos de la mezcla de reacción y cubrir con papel aluminio.
      3. Trasladar al área de procesamiento en cadena de frío y almacenar en refrigeración 2 a 8 °C, hasta el cargado del ARN:

**Área de procesamiento LVR NBS-II**

- Este proceso se realiza en el Laboratorio de Virus Respiratorio.
- Mezclar suavemente con micropipeta los ARN extraídos o agitador de tubos y agregar 5 μL de ARN extraídos al mix de PCR según corresponda, siguiendo el orden del protocolo de trabajo. (Volumen final 20 μL).
- Agregar 5 μL del control negativo (CN).
- Agregar 5 μL del control positivo (CP).
- Cerrar firmemente la tapa de cada tubo de PCR y colocar en el rotor del termociclador.

**Área Amplificación por PCR LVR NBS-II**

- Programar el termociclador según, la temperatura, ciclaje y el tiempo requerido que se detalla a continuación por cada uso de reactivo:
- Nota- El Panel 2 solo se preparará si el resultado en el Panel 1 sale POSITIVO y se deberá utilizar las áreas de procesamiento y amplificación como indica líneas arriba.
- Para el reactivo Biotechrabbit utilizar el siguiente programa de amplificación.

| Fases | Temperatura | Tiempo | Ciclos |
| --- | --- | --- | --- |
| Transcripción reversa | 50°C | 10 minutos | 1 ciclo |
| Denaturación inicial | 95 °C | 3 minutos | 1 ciclo |
| Amplificación por PCR | 95 °C | 10 segundos | 45 ciclos |
|  | 58 °C | 30 segundos |  |
|  | 40 °C | 30 segundos |  |

Adquirir en la etapa de hibridación en los canales Green y Orange a 58°C. Utilizar la opción de optimización de la fluorescencia durante el primer ciclo.

- - - 1. Al finalizar el programa, eliminar los tubos de reacción y realizar la limpieza la parte interna del termociclador.

**Lectura de Canal para:**

**Coronavirus-CoV-gen RdRP:**

- Green (470nm-510nm) FAM,Verde (green): Permite leer- CoV-19.

**GAPDH:**

- Orange (585nm-610nm) CAL filtro Fluor Red 610, anaranjado (orange): Permite leer -GAPDH humano.

# **CÁLCULOS:**

# “No aplica”.

# **INFORME DE RESULTADOS**

- Muestra Positiva: valor de Ct ≤ 37 se reportará como Covid-19.
- Muestras negativas: valor de Ct > 37 y un Ct del control interno menor o igual a 40 se reportará.
- Aquellas muestras que tengan valor del control interno mayor a 40 o no detectable se reportarán como: MUESTRA INDECUADA, (M.I).
- El analista registra los resultados en el **FOR-CNSP-590: Registro de resultados para otros virus Respiratorios,** el registro es revisado por un profesional autorizado por el responsable de laboratorio, quien deja evidencia de la revisión del registro colocando un check en la columna de resultado final, (si hubiera alguna observación se comunica al analista quien toma las acciones correspondientes). Luego, se entrega el registro de resultado al analista para el ingreso en el sistema informático NetLab. Finalmente, el personal autorizado para la revisión realiza la validación en el sistema Netlab dejando evidencia de la acción en la columna número del **FOR-CNSP-590: Registro de resultados para otros virus Respiratorios**

# **INTERPRETACIÓN DE RESULTADOS**

1. **El Control NTC,** no debe exhibir curvas de crecimiento de fluorescencia que crucen la línea de valor umbral.
2. **Los Controles Positivo (CP)** deben exhibir curvas de crecimiento de fluorescencia que crucen la línea de umbral en los primeros *35* ciclos. Se debe cambiar de control si este supera los 35 ciclos. Si no se visualiza amplificación en el control positivo se puede deber por problemas en los reactivos o en el funcionamiento de los equipos.
3. **El** **Control negativo (CN)**, no deben exhibir curvas de amplificación para Covid-19 que crucen la línea umbral en los primeros 40 ciclos.
4. **Se considera una muestra positiva** cuando la curva de crecimiento cruza el umbral en los primeros 35 ciclos en los sets de cebadores Covid-19.
5. **Se considera una muestra negativa**, cuando NO se evidencian curvas de crecimiento de fluorescencia que crucen la línea de valor umbral en los primeros 35 ciclos.
6. **Control interno (GAPDH**) deben exhibir curvas de reacción que crucen la línea umbral en los primeros 40 ciclos, indicando así la presencia de RNA del gen **GAPDH**. Este es un control que permite estimar si la cantidad de ARN en la muestra clínica es adecuada.

La imposibilidad de detectar curvas de amplificación en el control interno en cualquiera de las muestras clínicas puede ser por algunas de las razones que se indican:

• Extracción inapropiada de ácido nucleico de las muestras clínicas.

• Ausencia de células por deficiente obtención de muestra.

# **FORMULARIOS**

# FOR-CNSP-104: Protocolo de trabajo para extracción de ARN del virus SARS-CoV-2 por shock térmico.

FOR-CNSP-590: Registro de resultados para otros vírus respiratorios.

FOR-CNSP-095: Control de uso de equipos.

# **CONTROL DE CAMBIOS**

# “No aplica”.

# **ANEXOS**

Anexo 01: Reconstitución y preparación de cebadores y sondas de trabajo.

Anexo 02: Anexo 5. Purificación de ARN viral usando kit comercial de la marca GENEAID

**ANEXO 01**

**RECONSTITUCIÓN Y PREPARACIÓN DE CEBADORES Y SONDAS DE TRABAJO**

**RECONSTITUCIÓN DE CEBADORES Y SONDAS**

1. Con base en el número de nmoles de cada cebador o sonda calcular el volumen de agua.

2. Multiplicar por 10 el número de nmoles y agregar esa cantidad de agua al tubo liofilizado.

3. La concentración final será de 100µM (solución madre).

**PREPARACIÓN DE SOLUCIÓN DE TRABAJO**

**CEBADORES Y SONDAS PARA PANEL 1 (COV) y PANEL 2 (COVID-19)**

100 µM (solución madre) x **X** = 10 µM (concentración de trabajo) x 100 µL (volumen que se desea preparar)

**X** = 10 µL del cebador o sonda

Completar a 100 ul de agua de PCR

*realizar este procedimiento para cada primer y sonda liofilizado.

NOTA: en el caso de los primers y sondas de RNasaP, estos ya vienen reconstituidos a 10 µM (Solución de Trabajo)

**RECONSTITUCIÓN DE CEBADORES Y SONDAS gen GAPDH**

1. Con base en el número de nmoles de cada cebador o sonda calcular el volumen de agua.

2. Multiplicar por 10 el número de nmoles y agregar esa cantidad de agua al tubo liofilizado.

3. La concentración final será de 100µM (solución madre-1).

4. Hacer una segunda dilución para llevar a 50 µM (solución madre-2):

- En un vial estéril añadir 50 µL de la solución madre-1 y 50 µL de agua del PCR

**PREPARACIÓN DE SOLUCIÓN DE TRABAJO**

**SONDAS PARA Gen GAPDH**

50 µM (solución madre-2) x **X** = 1.25 µM (concentración de trabajo) x 100 µL (volumen que se desea preparar)

**X** = 2.5 µL del cebador o sonda

Completar a 100 ul de agua de PCR

**CEBADORES PARA Gen GAPDH**

50 µM (solución madre-2) x **X** = 2.5 µM (concentración de trabajo) x 100 µL (volumen que se desea preparar)

**X** = 5 µL del cebador o sonda

Completar a 100 ul de agua de PCR

**ANEXO 2**

**PURIFICACIÓN DE ARN VIRAL USANDO KIT COMERCIAL DE LA MARCA GENEAID**

Debido a la necesidad de evaluar otras marcas comerciales se estandarizo y evaluó la purificación de ARN viral usando el kit comercial de la marca BiotechRabbit. Viral Nucleic Acid Extraction Kit II de la marca GENEAID.

- Rehidratar el carrier ARN con 1 mL de agua libre de ADNsas y ARNsas (provista en el kit) respectivamente. Luego de hidratados este reactivo mantener en congelación (-20 °C). Añadir la cantidad requerida (indicada en los frascos de los reactivos) de etanol absoluto a la solución AD buffer y al buffer de lavado WASH buffer.
- Calcular y añadir la cantidad necesaria de buffer de lisis (400 µL por cada muestra) y carrier (4 µL por cada muestra) en un tubo cónico de 15 mL estéril.
- Añadir 400 µL de solución de lisis/carrier a un tubo de 1.5 mL limpio y estéril.
- Añadir 200 µL de muestra al tubo indicado en el paso anterior.
- Homogenizar completamente mediante pipeteo (pipetear 5 veces) o con el agitador de tubos de 10 a 15 segundos.
- incube a temperatura ambiente durante 10 minutos.
- Añadir 450 µL de solución AD buffer a cada tubo y mezcle completamente durante 10 segundos. Centrifugue el tubo brevemente.
- Añadir 600 µL de la mezcla a la columna de purificación y centrifugar durante 1 minuto a 14000 r.c.f. Eliminar el tubo colector.
- Coloque la columna en un tubo de colección nuevo. Repetir el paso anterior en el resto de la muestra.
- Colocar la columna de purificación en un nuevo tubo recolector. Añadir 400 µL de solución de lavado W1 buffer. Centrifugue durante 30 segundos a 14000 r.f.c. Eliminar el tubo colector.
- Colocar la columna de purificación en un nuevo tubo recolector. Añadir 600 µL de solución de lavado WASH Bufer. Centrifugar durante 1 minuto a 10000 r.f,c. Eliminar el tubo colector.
- Para remover completamente la solución de lavado remanente en la columna colocarla en un tubo de colector nuevo y centrifugar por 3 minutos a 14000 r.f,c. Eliminar el tubo colector.
- Colocar la columna de purificación en un vial de 1,5 mL estéril rotulado. Cuidadosamente abrir la tapa de la mini columna insertada en el vial y añadir 50 µL de agua libre de ADNasas y ARNasas (proceso conocido como elusión). Incubar a temperatura ambiente por 3 minutos y centrifugar durante 1 minuto a 14000 r.f.c.
- El ARN extraído se almacena en el refrigerador de 2 a 8 °C hasta continuar con la prueba molecular de PCR. En caso de no utilizar ese mismo día se conserva de -20 a -70 °C para conservar la estabilidad del ARN.
