# Supplementary material for: A faster and less costly alternative for RNA extraction of SARS-CoV-2 using proteinase k treatment followed by thermal shock
Source: PLoS One. 2021 Mar 24;16(3):e0248885. doi: 10.1371/journal.pone.0248885 (PMC7990203; doi:10.1371/journal.pone.0248885)
Supplement: Attachment — Submitted filename: Reponse to the editor and reviewers.docx [file pone.0248885.s004.docx]

**REPONSE TO THE EDITOR AND REVIEWERS**

**PONE-D-20-28949**

**#EDITOR:**

Please clarify whether this [conference proceeding or publication] was peer-reviewed and formally published. If this work was previously peer-reviewed and published, in the cover letter please provide the reason that this work does not constitute dual publication and should be included in the current manuscript.

**R: The data related to the standardization of RT-qPCR cited in our paper is from the INS and only was used as a useful tool to test our RNA extraction method. Since these data are from INS, this does not constitute a dual publication.**

**Reviewer #1:** In this study, the authors compared the performance of proteinase K treatment followed by thermal shock with commercial extraction methods, which provides an alternative way to test SARS-CoV-2 in low income regions. The comments discussed below are provided for consideration.

1. The authors used three different enzymes and reagents to perform PCR, but didn’t mention the differences between these reagents. Could the discrepancy between results be due to the reagents used?

**R: The enzymes and reagents mentioned were used in the preliminary tests, considering the standard procedures for implementing a new INS protocol (data not relevant). The Biotech Rabbit was used in the RT-qPCR reactions to compare only the extraction methods. Since we conducted new RT-qPCR reactions for replicates, we only used a type of enzyme, the Biotech Rabbit, to standardize the reactions and consider only the comparison between the RNA extraction methods.**

2. Table 3: How many times was each sample repeated? Where does average and SD come from?

**R: We conducted new RT-qPCR reactions in triplicate. The new data were updated and submitted as table S1.**

3. As seen in Table 3, although the statistical analysis of variance showed no significant difference, the CT value of several samples is largely different in commercial kits group and PK+TS group. Such as 84667, 84677, 85480, 85481…

**R: The new statistical analysis showed a significant difference between RNA extracted using our method compared to that used commercial kit. Also, we identified higher variance between samples when extracted by commercial kit than that extracted using our method. This point could be justified by the column method that uses solid phase and can saturate. Since the internal control is more abundant than viral RNA and our method considers total RNA in the solution, the saturation does not occur, and the RT-qPCR reactions can detect the GAPDH RNA more easily (this material is widely available).**

4. As for testing RNA virus, RNaseP might be better than GAPDH as internal control.

**R: We agree with this point. The GAPDH gene was already used as an internal control for the detection of SARS COV2. Besides, the supplies were available, and their cost is lower compared to commercial RT-PCR kits.**

5. Line 283, is it possible that proteinase K treatment caused false positive result? The inconsistent results should be confirmed with a more sensitive reagent. Also, the successful amplification of GAPDH cannot guarantee that there were no false negative results.

**R: We performed the RNA extraction from the same sample using both methods, and the results were 100% in agreement. In this sense, the samples with low viral load should present disagreement results between the methods (the commercial kit should be more sensible), which was not valid. We understood that our protocol presented the same efficiency as that commercial kit to extract the SARS-CoV-2 RNA.**

6. Table 4, the positive rate of week 24 and 25 is slightly lower than other weeks.

**R: Even the positivity data presented in the epidemiological weeks 24 and 25 was slightly lower than the others. There was none significant difference between them.**

**Reviewer #2:** This article is timely in the current pandemic, with many readers likely keen to learn as much about COVID-19 as possible. However, there are some major issues with this article.

**Title: suitable**

**Abstract**: The abstract should contain a mention of the specific study design and at the end should contain a sentence on implication of this study.

**Background: The background is adequate**

**Methods**: The methods section should be revisited. Is this indeed a **retrospective study**? In a retrospective study of how the two methods were compared. How was the sample compared before and after archiving? What is the commercial kit used in this study?

**Results**: The results are adequate for a retrospective study

**Discussion**: There is need to discuss these findings to reflect the objectives of the study. Authors should focus on these and compare their results critically with other similar studies and derive a valid inference based on their findings.

**Conclusion**: adequate for a retrospective study

**References**: inadequate

**General comments**: The whole manuscript needs copy-editing and should preferably be **edited by an English expert or native English speaker**

**Reviewer #3:** The presented manuscript by Ñique and colleagues presents a helpful addition to the growing list of publications describing alternatives to kit-based RNA isolation for SARS-CoV-2 diagnostics, which is of continuing importance considering increasing case numbers in secondary waves of infection in countries with limited resources. Their side-by-side comparison of kit-based and kit-free RNA preparation with SARS-CoV-2 and endogenous control target detection is sound and shows that ProtK + Heat shock is a suitable alternative to RNA purification if resources are limited. However, **the article suffers from poor English in some places, making it less comprehensible**. The presented method should also be compared to already published kit-free protocols for SARS-CoV-2 qPCR rather than unrelated protocols for DNA processing. **Some conclusions need to be revised – there cannot be 100% concordance between the protocols if at least 1/72 samples are positive in one protocol but not the other, as discussed**. Comparing epidemiological weeks when kit-based and kit-free detection was performed based on positivity rates is interesting, but it should be added that this cannot be proof of whether the method is superior/equivalent or not, as there is no direct comparison for the same samples. A revised version would be suitable for publication in PLOS ONE.

Typo in financial disclosure “associated whit Ministry of Health”

Language - in competing interests “declare none” – should be “no”

**Line 1:** Title “**faster**” – than what? Change to fast or include “kit-based” or something like this

**R: The title was updated according to your suggestion.**

**Line 26**: Change sentence structure “Sometimes, even with the funds and resources available to purchase them, the lack of suppliers and the global shortage of material as consequence of the high demand, makes timely detection and diagnosis of the virus carrier difficult.”

**R: Sometimes, the lack of suppliers and the global shortage of material due to the high demand make it difficult for timely detection and diagnosis of the virus carrier even with the funds and resources available to purchase the reagents.**

**Line 34**: Concordance was 100% and then RT-qPCR was performed” – concordance with test samples? Add this, otherwise confusing.

**R: The concordance between results obtained by RT-qPCR from both RNA extraction methods was 100% in the samples tested. In the data presented in table 3, there was a disagreement related to only one sample. Since one of the reviewers suggested that we present results obtained from average, we executed new rounds of experiments, considering experimental triplicate and including other six samples more (from 72 samples to 78 samples; from unique running to triplicate running). The new results presented a 100% in agreement.**

**Line 37**: This sentence is confusing and only made sense after reading the entire manuscript, so it should be formulated more clearly. I would suggest the following: “**We compared the SARS-CoV-2 positivity obtained using our kit-free method within two complete epidemiological weeks to the two weeks prior and after, where RNA was extracted using commercial kits. There was no significant difference between positivity rates, suggesting the use of a kit-free RNA preparation method did not significantly alter detection rates**.”

**We make all changes according to the suggestions.**

**Line 41**: Language – “**there was no significant difference**”. Also, what does “**positive results’ average**” mean?

**Line 49**: “**type**” is not part of the name of the virus, it’s just “Severe acute respiratory syndrome coronavirus 2”

**Line 54**: Language – change to “**and makes them**”

**Line 65**: **5-log reduction in what – infectivity? Please add**.

**R: The information was added.**

**Line 66**: Language – change to “**are also used during amplification by RT-qPCR**”

**Line 67**: Language – change to “**Furthermore, using an internal control for RT-qPCR reaction can confirm integrity of the samples, decreasing the probability of false-negative results**” (“exponentially” is odd here)

**Line 70**: There’s something missing in this sentence. I assume it should be something like “… **physical methods like heating and specific chemical reagents like proteinase K widely used in genetic material extraction protocols may be used to optimize viral purification**”

**Line 76**: Remove “**~~assay~~**” – a purification is not an assay.

**R: The word was removed.**

**Line 77**: Spelling “stablished” – change to “**established**”

**Line 79**: Language – change to “**We compared to efficiency of the new method based on Ct values obtained by**…”

**Line 91**: Language – I assumed “**~~people suspected~~**” should just be “**patients**”

**Line 92**: Language – change to “**sample collection**”

**Line 132-147**: This paragraph is confusing. You tested 72 samples with both methods. Also, during June 3-25, all diagnostics was done with the presented ProtK/thermal shock method because no kits were available.

1. Was this before or after the 72 samples were tested with both methods?

**R: These proceedings for COVID-19 molecular diagnosis using RNA extracted using heat shock and proteinase k were conducted after validation using the 78 samples.**

2. Then you’re comparing positivity obtained in the period where kit-less methods were used to other periods where kit-based methods were used – on the assumption that numbers would be similar based on progression (or rather non-progression) of the pandemic? This is interesting, but should not be over-interpreted and treated with caution.

**R: The sentence was rewritten considering this caution.**

**Line 181**: Language – “**in both channels**”

**Line 198**: Here you write that ANOVA was used on Ct values, while in the methods section (line 181) you write that “**qualitative results of RT-qPCR were submitted to ANOVA**”. Ct is a quantitative result.

**R: We conducted experiments without using a standard curve. In our case, the Ct values were only indirect measures of viral load.**

**Line 202**: You should discuss why GAPDH is significantly more abundant in the non-kit samples. The differences are quite substantial – I would suspect this is from cellular debris that may be more abundant in the non-kit-purified samples.

**Line 223**: Language/confusing – there was an increase in cases (and testing), likely due to the lockdown being lifted but the increase was not significant compared to previous weeks? If so, rephrase to “**this change in behavior in the population apparently didn’t lead to a significant increase in positivity**”.

**Line 226**: Spelling “**~~stablishing~~**” – change to **establishing**.

**Line 229**: Language – change “**~~provided~~**” to “**made it necessary to implement and validate protocols in house**…”

**Line 232**: Language – change to “**is essential to make it possible to process sufficient samples in underdeveloped countries**”

**Line 234**: Language – “**sample quality**”

**Line 239**: It’s okay to mention this but DNA from spores and RNA from viruses are hardly comparable, so I would remove some of the details that don’t add anything to this manuscript.

**Line 254**: Language – “since the samples are processed in 96 well plate format”. Also, especially since this is a crucial point, just state the time/sample. Currently it sounds odd that 5.5h is “8 times higher” than “less than 1h”.

**Line 257**: Similar to the spores, not really relevant to compare environmental DNA to viral RNA.

**Line 264**: It is also okay to cite preprints. Besides there are multiple published manuscripts describing “kit-free” RNA preparation for qPCR of SARS-CoV-2 that should be discussed.

These papers should be included in the discussion.

<https://doi.org/10.1016/j.jviromet.2020.113965>

<https://doi.org/10.3390/diagnostics10080605>

<https://doi.org/10.1038/s41598-020-73616-w>

<https://doi.org/10.1371/journal.pbio.3000896>

<https://doi.org/10.1371/journal.pone.0236564>

<https://doi.org/10.1038/s41467-020-18611-5>

<https://doi.org/10.1016/j.jcv.2020.104423>

**R: All these papers suggested were included in the discussion section.**

**Line 274**: Language – “**since these variables**”

**Line 276**: Why the quotation marks? Also its rather what makes this “purification” possible, not an advantage.

**R: The quotation marks was removed.**

**Line 283**: Earlier in the manuscript you write about “100% concordance”, yet here is a sample where this is not the case. I would also caution the interpretation that a sample negative by definition of Cut-off when purified with the commercial kit but positive when treated with ProtK/thermal shock was “false-negative” with the commercial kits – both values are very high and I would be cautious about higher background signal in non-purified samples.

**R: Here we can explain that the RT-qPCR protocol used in our study was submitted to the validation process, and the positivity was measured from this point, e.g., from the cut-off obtained in that validation. There was no relation to the results presented in our paper about heat shock and proteinase k for RNA extraction.**

**Line 291**: But the advantage of multiplexed detection of SARS-CoV-2 and an endogenous control would be the case for both the commercial kit and your in-house method. And you cannot exclude false-positives by your method compared to commercial kits based on higher background.

**Line 292**: “Our results showed no significant difference between Ct values obtained when the same sample were processed in parallel by both RNA extraction” – what about **sample 84677** mentioned just in the above paragraph?

**Line 294**: “Ct values of positive control have always been in agreement” – but this is already isolated RNA. Did you also subject the sample to your in-house method of ProtK/Heat?

**R: In this case, we think this point is not necessary because positive control is a standard. Many researchers use g-block or other synthetic genetic material as a positive control in their RT-qPCR reactions. Furthermore, the experiments were conducted using clinical samples, which should be considered more critical.**

**Line 301**: Would be highly cautious calling something a “better yield” based on one sample where this happened (see above comment)

**R: The expression was removed.**

**Table 2:** Add the actual product used somewhere (catalogue ID).

**R: This information was updated.**

**Table 3:** It would be helpful if this was also displayed graphically. Also please state what the averages are derived from – commercial kit and PK + TS?

**R: This table was removed and submitted as support material (table S1).**
