# Supplementary material for: A faster and less costly alternative for RNA extraction of SARS-CoV-2 using proteinase k treatment followed by thermal shock
Source: PLoS One. 2021 Mar 24;16(3):e0248885. doi: 10.1371/journal.pone.0248885 (PMC7990203; doi:10.1371/journal.pone.0248885)
Supplement: Attachment — Submitted filename: Response to Reviewers-Alternative method for RNA extraction.docx [file pone.0248885.s005.docx]

**Response to Reviewers**

Please authors, you must be replied to the reviewer comments. The manuscript should be revised by expertise in English language.

R: The authors carefully replied to all questions from the reviewers. In addition, the protocol is now available in <https://doi.org/10.17504/protocols.io.br6bm9an>.

**Response to the Reviewer #3:**

1. The revised manuscript is improved in several areas, but there are still many language problems. The manuscript needs proof-reading by someone proficient in English. I will only point out the major language problems specifically here. Scientifically, the discussion is greatly improved but would benefit from more careful examination of the cited literature and less exaggerated conclusions regarding superiority of the presented method.

R: The paper was submitted to a careful review by a native English professor, and the discussion and the conclusions were restructured accordingly, suggested by the reviewer.

2. The title already has odd sentence structure.

R: The paper title was changed to “A faster and less costly alternative for RNA extraction of SARS-CoV-2 using Proteinase k treatment followed by thermal shock”.

3. Abstract:

“the variance was higher in the samples extracted by commercial kit than that used the alternative protocol, suggesting indirectly that our protocol was more efficient to total RNA extraction”.

R: This sentence was deleted.

4. The misinformation about the experimental design of Michel and collaborators (2020) was eliminated.
5. The term “was really good” was deleted.
6. The sentence “Common RNA purification kits have, in contrast, a step for the removal of genomic DNA. Our non-kit-purified protocol does not contain any DNase step, resulting in higher amounts of cellular debris and contaminating DNA in the solution. The extra material is not a problem for the viral targets but can explain significant variations in *GAPDH* detection", according to the reviewer's suggestion.
7. The sentence “Our protocol, additionally to solve our temporary” was deleted.
8. The sentence “More than a new method, our protocol seems to be a large vantage as a public health approach” was deleted.

**Response to the Reviewer #2:**

1. Questions and answers related to the new round of experiments.
a) Are these new samples?

R: The experiments were repeated under the same conditions, using the same range of Ct values and in experimental triplicate, but using a new round of samples. This change occurred because either the original samples were no longer available in our sample-bank or the RNA was degraded, preventing the new experiments, according to the reviewer's suggestion.

b) Why is the numbering different than in the initial manuscript?

R: Seeing we had to conduct new experiments for guaranteeing our protocol yield and considering the discordant result presented in sample 84677, we included six more samples (almost 10% increase). Unfortunately, sample 84677 was no longer available and therefore not included in the experimental replicates. I would suggest showing the comparison of Ct obtained by both kits as a dot plot with regression line, so one can immediately see how similar it looks (for both the viral and control target).

R: The dot plot with the regression line related to the comparison of the Ct values (viral target – *RdRp* gene: RNA extracted by our protocol versus RNA extracted using commercial kit; internal control – *GAPDH* gene: extracted by our protocol versus RNA extracted using the commercial kit) was produced and is now available bellow.

2. Then you’re comparing positivity obtained in the period where kit-less methods were used to other periods where kit-based methods were used – on the assumption that numbers would be similar based on progression (or rather non-progression) of the pandemic? This is interesting but should not be over-interpreted and treated with caution.

R: The sentence was rewritten considering this caution.

3. I don’t see any changes reducing the conclusions on this. The manuscript submitted is also not fully change-tracked, as there are changes here but none changing the conclusions.

R: The conclusion was rewritten according to both reviewers' suggestions, and the file was carefully change-tracked.

4. This is fine, but Ct is still a quantitative measurement. You’re not comparing “positive vs. negative” in the ANOVA, so it’s still a comparison of quantitative data.

R: The word “qualitatively” was deleted.

5. Would be highly cautious calling something a “better yield” based on one sample where this happened (see above comment) R: The expression was removed. Reviewer response: Not really, you’re still making the same point in Line 408 as mentioned above. I will believe this if you show it’s not DNA contamination; but you can also just tone down this conclusion.

R: The idea was restructured in all the papers, and these cautious were carefully considered by the authors.
